# Contribution of cod liver oil-related nutrients (vitamins A, D, E and eicosapentaenoic acid and docosahexaenoic acid) to daily nutrient intake and their associations with plasma concentrations in the EPIC-Norfolk cohort

**DOI:** 10.1111/jhn.12271

**Published:** 2014-09-16

**Authors:** M A H Lentjes, A A Mulligan, A A Welch, A Bhaniani, R N Luben, K–T Khaw

**Affiliations:** 1 Department of Public Health & Primary Care, EPIC-Norfolk Study, University of CambridgeCambridge, UK; 2 Norwich Medical School, Department of Population Health and Primary Care, University of East AngliaNorwich, UK; 3 University of Cambridge, School of Clinical Medicine, Clinical Gerontology UnitCambridge, UK

**Keywords:** biomarkers, cohort study, dietary assessment, dietary supplements, total nutrient exposure

## Abstract

**Background:**

Total nutrient intake (TNI) is intake from food and supplements. This provides an assessment of nutrient adequacy and the prevalence of excessive intake, as well as the response with respect to biomarkers. Cod liver oil (CLO) is the most frequently consumed supplement in the UK, containing nutrients that might have varying influences on health. We calculated TNI for vitamins A, D and E, as well as eicosapentaenoic acid (EPA) and docosahexaenoic acid (DHA), and assessed associations with the respective blood concentrations.

**Methods:**

Seven-day diet diaries and blood samples were taken from two subsets of the European Prospective Investigation into Cancer (EPIC-Norfolk) cohort (age range 39–79 years; *n* = 1400 for vitamin D; *n* = 6656 for remaining nutrients). TNI was calculated for the subgroups: nonsupplement users, those consuming the nutrient in supplement form and those consuming a supplement without this nutrient.

**Results:**

CLO-related nutrients were supplemented by 15%–33%, which approximately doubled median intakes. Almost everyone in the supplement + vitamin A group reached the estimated average requirement; however, guideline levels were likely to be exceeded. Partial correlations between intake of vitamins A and D and biomarkers were low and modestly strengthened by the inclusion of supplement sources (correlation = 0.01–0.13). Correlations between biomarker and TNI of vitamin E and EPA+DHA were in the range 0.40–0.46; however, vitamin E exceeding food intake resulted in attenuated coefficients. Linear associations between food or TNI EPA+DHA and plasma were weak but consistent across subgroups.

**Conclusions:**

CLO-related nutrients contribute substantially to nutrient intake, with a risk of over-consumption. Apart from EPA+DHA, biomarker data suggest that CLO-related nutrients in supplements are not linearly associated with vitamin status.

## Introduction

In the European Prospective Investigation into Cancer and Nutrition in Norfolk (EPIC-Norfolk), a population-based cohort in the UK, dietary supplements are used by 32% of men and 45% of women (Lentjes *et al*., 2013), with cod liver oil (CLO) being consumed most frequently (Lentjes *et al*., 2011). The Vitamin and Mineral Supplement (ViMiS) database and software that have been developed enable the assessment of combined food and supplement intake into total nutrient intake (TNI) (Lentjes *et al*., 2011). Knowing the TNI has three advantages. First, a comparison of TNI with average requirements and upper limits can better identify groups at risk of inadequate or excessive intakes. For example, the potential harmful aspects of an excessive intake of vitamins A and D can only be assessed when both food and supplement sources are quantified (Expert group on vitamins & minerals, 2003). Second, associations with nutrient intake and blood biomarkers could become stronger and provide better insight into the diet-biomarker-disease association. Third, associations of nutrient exposure and biomarkers are not linear (e.g. vitamin E) (White *et al*., 2001; Lebold *et al*., 2012). The association is not apparent unless the full range of intake is assessed; for example, by including supplement sources (Vogel *et al*., 1997).

UK studies (Kirk *et al*., 1999; McNaughton *et al*., 2005) report that supplement users (SU) have higher energy, macro- and micronutrient intakes from food alone than nonsupplement users (NSU) and the National Diet and Nutrition Survey (NDNS) shows that, at the population level, supplement use has an impact on the proportions reaching the reference nutrient intake (Henderson *et al*., 2003). However, a comparison of all SU together, disregarding the type of supplement, and hence which nutrient a SU adds to their diet, may introduce errors in the exposure measurements. This might be particularly important where the range of safe nutrient intake is relatively narrow (e.g. for retinol) (Mulholland & Benford, 2007).

Cohort studies relating CLO-related nutrient intake to biomarker data have reported that vitamin E intake has positive correlation coefficients with plasma α-tocopherol, ranging from 0.40 (food source) to 0.69 (TNI) (Vogel *et al*., 1997; White *et al*., 2001; Satia-Abouta *et al*., 2003; Lebold *et al*., 2012), whereas it has negative correlations with γ-tocopherol, ranging from −0.04 (food) to −0.54 (TNI) (Vogel *et al*., 1997; White *et al*., 2001; Lebold *et al*., 2012). Trial data show associations between intake and respective blood biomarkers of eicosapentaenoic acid (EPA) and docosahexaenoic acid (DHA) (Arterburn *et al*., 2006). Spearman correlations between TNI and plasma in observational studies were in the range 0.53–0.58 (Hjartåker *et al*., 1997), with lower correlations between food sources only and plasma in the range 0.23–0.29 (Astorg *et al*., 2008). Although 25–hydroxy cholecalciferol is mainly produced in the skin under influence of sunlight, a 10 nmol L^−1^ higher concentration has been found among participants using low-dose (5 μg day^−1^) supplements (Hyppönen & Power, 2007). In the UK, where CLO is the most common dietary supplement (Henderson *et al*., 2002), all of these nutrients are provided in a single capsule, raising concerns about collinearity when aiming to examine associations between single nutrients and outcomes (Patterson *et al*., 1997), although this also enables the assessment of possible synergistic effects on plasma concentrations (e.g. omega-3 fatty acids and vitamin E) (Institute of Medicine, 2000).

The analyses extend on the analogy used by Block *et al*. (1994) regarding the quantification of cigarette smoking and nutrient intake: one would not calculate the average amount of cigarettes smoked for the population as a whole, rather only that for smokers. Hence, rather than comparing all SU versus NSU, we describe the contribution of CLO and nutrient-related supplements to nutrient exposure by the nutrient supplemented and the effect that supplements have on the prevalence of adequate and excessive nutrient intake. Additionally, we analysed the association between nutrient intake, from both food and TNI, and respective blood biomarkers as surrogate indicators of absorption and metabolism in a population where only a minority use single, high-dose supplements.

## Materials and methods

### Study design

EPIC-Norfolk is a prospective cohort study into the determinants of health and chronic diseases (Day *et al*., 1999). The study was approved by the Norwich District Health Authority Ethics Committee and participants provided their written informed consent. The first round of data collection started in 1993 and finished in 1998. The study recruited 30 445 participants, of whom 25 639 attended a health examination at their general practitioner's practice. A trained nurse measured participant's height (cm) and weight (kg) and obtained a blood sample.

### Blood analysis

A 42-mL sample of blood was taken in nonfasting state (Day *et al*., 1999). Blood was collected in citrated and plain monovettes and stored in a refrigerator. The next day, blood samples were processed and stored at −196 °C as plasma and serum. Serum cholesterol was determined for the full cohort in a Norfolk laboratory using a RA 1000 Diagnostics (Bayer, Basingstoke, UK) instrument, and cohort concentrations ranged from 2.10–12.40 mmol L^−1^. The remainder of the blood samples were analysed on subsets of participants (Table 1). Serum 25-hydroxy cholecalciferol [25(OH)D3] and ergocalciferol [25(OH)D2] were determined as part of a nested case–cohort study that consisted of 892 participants with incident type 2 diabetes and a random sample (*n* = 989) of the EPIC-Norfolk cohort (Forouhi *et al*., 2012). Ultra-performance liquid chromatography-tandem mass spectrometry was used to measure serum concentrations, using a standardised assay. Measured concentrations ranged from 9.48–163.74 nmol L^−1^ for 25(OH)D3 and from 0.25–24.21 nmol L^−1^ for 25(OH)D2 [we excluded both concentrations for one participant who had a 25(OH)D2 concentration of 45 nmol L^−1^]. The vitamins retinol and α- and γ-tocopherol, as well as the omega-3 fatty acids EPA and DHA, were analysed on a participant subset that consisted of a series of previous case–control studies, where cases were defined by incident cardiovascular disease or cancer and four matched, disease-free controls. Plasma concentrations were analysed at IARC, Lyon (France) using high-performance liquid chromatography for the vitamins [concentrations in the range: retinol 0.27–5.29 μmol L^−1^, α-tocopherol 3.36–100.58 μmol L^−1^ and γ-tocopherol 0.07–9.54 μmol L^−1^; we excluded one participant for whom γ-tocopherol was greater than α-tocopherol (ratio <1), as well as two participants where this ratio was >1000 (where the remainder of the ratios reached a maximum of 522)]. Gas chromatography was used to analyse concentrations of the fatty acids (ranging from 47–1707 μmol L^−1^). EPA and DHA were summed, for both nutrient intake and plasma concentrations, aiming to avoid issues surrounding retroconversion of DHA to EPA (Arterburn *et al*., 2006).

**Table 1:** Characteristics of full cohort and participants included for the analysis on plasma vitamins A, E and eicosapentaenoic acid (EPA)/docosahexaenoic acid (DHA), as well as participants with available serum cholecalciferol

	Men			Women		
	Men cohort*	With plasma A, E, EPA and DHA	With serum 25(OH)D	Women cohort*	With plasma A, E, EPA and DHA	With serum 25(OH)D
*n* (% men/women)	11 607 (45.3)	3365 (50.6)	656 (46.9)	14 032 (54.7)	3291 (49.4)	744 (53.1)
Age (years), mean (SD)	59.1 (9.3)	64.1 (7.8)	60.5 (8.8)	58.4 (9.3)	61.6 (8.7)	59.4 (9.4)
BMI (kg m^–2^), mean (SD)	26.5 (3.3)	26.7 (3.3)	27.4 (3.6)	26.2 (4.3)	26.3 (4.2)	26.9 (4.5)
Cholesterol (mmol L^−1^), mean (SD)	6.04 (1.10)	6.09 (1.10)	6.03 (1.08)	6.30 (1.21)	6.49 (1.24)	6.41 (1.25)
Social class (% manual)	41.6	41.6	43.8	38.6	38.9	40.0
Education (% no qualification)	35.0	36.2	34.0	42.2	48.0	44.6
Smoking (% current)	12.2	10.6	10.6	11.4	10.2	13.2
Physical activity [% (moderately) inactive]	55.5	60.0	55.1	62.5	65.6	63.3
Season (% winter)	23.6	24.9	24.2	22.8	23.0	22.3
Supplement use (% yes)	31.7	32.6	29.4	44.8	43.7	41.1
Self-reported heart attack (% yes)	5.3	7.9	8.9	1.3	2.2	1.8
Self-reported diabetes (% yes)	3.1	5.2	1.8	1.6	2.8	1.2
Self-reported cancer (% yes)	3.8	4.2	2.9	6.8	5.7	7.3
7-day diet diary:						
Energy (MJ day^−1^), mean (SD)	9.45 (2.22)	9.17 (2.07)	9.35 (1.98)	7.15 (1.66)	7.10 (1.58)	7.19 (1.65)
Fruit (g day^−1^), mean (SD)	158 (134)	164 (127)	158 (136)	183 (133)	189 (133)	189 (133)
Vegetables (g day^−1^), mean (SD)	151 (79)	152 (76)	151 (74)	150 (76)	149 (73)	147 (69)
Fatty fish (g day^−1^), mean (SD)	13 (23)	14 (22)	14 (24)	12 (18)	12 (17)	12 (17)
Fatty fish (g day^−1^)†, mean (SD), consumers only	27 (26)	26 (24)	27 (28)	20 (20)	21 (18)	22 (18)

25(OH)D, 25-hydroxy cholecalciferol and ergocalciferol; BMI, body mass index.

* Participants who attended the health examination at their general practitioner's clinic (*n* = 25 639).

† Proportion (%) of men who consumed fatty fish during their 7-day diet diary period: 49%, 53%, 53%, and among women: 52%, 55% and 54%.

### Measuring dietary nutrient intake

The participants who came for a health examination received a 7-day diet diary (7dDD). A nurse explained the purpose of the 7dDD and took a 24-h diet recall to ensure good quality of recording by the participant for the remaining 6 days. The days were divided into eight different potential meal occasions. Portions could be quantified using household measures, packaging or any of the 17 coloured photos. The 7dDD was completed by 25 507 participants, with 23 638 (92%) completing more than 1 day. They were entered into bespoke software [Data Into Nutrients for Epidemiological Research (diner)] (Welch *et al*., 2001) and checked by nutritionists for completeness and consistency. Nutrient outliers were identified and data-entry errors were corrected (Lentjes *et al*., 2014).

### Measuring supplement nutrient intake

Supplements were recorded by participants in the Back of the Diary (BOD) (Lentjes *et al*., 2011). The ViMiS database contained the nutrient analyses per unit of consumption using supplement-specific manufacturers’ data. Where the participants did not record the supplement data in sufficient detail, a weighted average of available options was taken from the ViMiS database. Missing values for EPA and DHA nutrient composition were estimated from CLO supplements of similar type (capsule versus liquid) and CLO content. Quantities of vitamins and fatty acids were converted to match the unit of food intake. The average daily nutrient intake from supplements for each participant was calculated and added to their average daily food intake to obtain TNI. Participants were grouped into one of the three subgroups:

- NSU: participants not consuming any supplement (the nutrient intake was derived from food sources only);
- SU−: participants consuming (a) supplement(s) but this supplement did *not* contain the specific nutrient being studied (the nutrient intake was derived from food sources only);
- SU+: participants consuming (a) supplement(s) that contained the specific nutrient being studied (the nutrient intake was derived from food and supplement sources); participants who consumed a supplement with a daily nutrient intake below the set thresholds (i.e. supplement intake below 5% of mean cohort intake from food sources only), were considered SU− rather than SU+, as described in detail elsewhere (Lentjes *et al*., 2011).

### Daily reference values

The daily reference values and upper levels for the vitamins studied were defined as:

- Estimated average requirement (EAR), used for comparing populations against a standard. It is the average nutrient requirement in a group (COMA, 1991; Carriquiry, 1999).
- Safe upper level (SUL), ‘represents an intake that can be consumed daily over a lifetime without significant risk to health on the basis of available evidence (Expert Group on Vitamins and Minerals, 2003)’
- Guidance level (GL), ‘an approximate indication of levels that would not be expected to cause adverse effect but have been derived from limited data and are less secure than SULs (Expert Group on Vitamins and Minerals, 2003)’

### Statistical analysis

Only participants who attended the health examination, who completed the BOD regarding dietary supplement use, and for whom serum cholesterol was determined, were included in the analysis (*n* = 1400 for vitamin D; *n* = 6656 for the remaining nutrients). Cholesterol was included, because plasma EPA, DHA and the fat-soluble vitamins were adjusted for cholesterol. The characteristics of the full cohort and the two subcohorts are given in Table 1.

Nutrient and plasma/serum concentrations were described by supplement subgroup (NSU/SU−/SU+). These data were positively skewed; hence, Mann–Whitney tests were used to test for differences in nutrient intake and biomarker data between the subgroups [*P* < 0.017 (0.05/3) was considered statistically significant]. The proportion in EPIC-Norfolk not meeting the EAR, or exceeding the SUL or GL, was estimated, with and without supplement sources. Similarly, proportions were given below the deficiency concentration (0.7 μmol L^−1^) of plasma retinol (Vogel *et al*., 1997); three different serum 25(OH)D3 concentrations reflecting rickets prevention (25 nmol L^−1^), common laboratory reference value for identifying low concentrations (40 nmol L^−1^) and concentrations suggested for optimal bone health (75 nmol L^−1^) (Hyppönen & Power, 2007); and plasma α-tocopherol concentrations needed to prevent hemolysis (12 μmol L^−1^) (Institute of Medicine, 2000).

The log_10_ transformed nutrient intake and its plasma concentration were plotted (Figure S1). Linear associations between intake and biomarker data were tested per 800 μg day^−1^ of retinol equivalent (RE) intake (common supplement dose), per 5 μg day^−1^ of vitamin D intake (common supplement dose), per 10 mg day^−1^ of α–TE (tocopherol equivalent) intake (common dose in CLO and multivitamins/multiminerals supplements) and per 100 mg of EPA and DHA intake (low-dose range in capsules). Partial correlations are given (i.e. associations between biomarker and nutrient intake from food only and TNI), adjusted for common confounders (Vogel *et al*., 1997; White *et al*., 2001; Arab, 2003). Vitamin D was adjusted for energy intake (per MJ), season (spring/summer versus autumn/winter), physical activity [(moderately) active versus (moderately) inactive], age (per 5 years) and body mass index (BMI) (per kg m^–2^). Concentrations for plasma tocopherol and EPA+DHA were log-transformed and these biomarkers, as well as retinol, were adjusted for energy intake, serum total cholesterol (per mmol L^−1^), BMI, smoking (current versus former/never), alcohol intake (per 8 g alcohol day^−1^) and age. Of the log-transformed biomarker data, the exponent of the β was taken; the β coefficient therefore represents a percentage change in plasma for a unit change in intake [e.g. for every 10 mg of vitamin E intake (*x*-axis), the mean percentage decrease in plasma γ-tocopherol that was observed (*y-*axis)]. Finally, the effect of the modification of omega-3 fatty acid concentrations by vitamin E intake was formally tested by inserting an interaction term of CLO supplements and vitamin E supplements using univariate analysis of variance (ancova). In other words, we tested whether we could observe an additional increment in plasma EPA+DHA when vitamin E was supplemented in combination with CLO, as opposed to CLO supplementation on its own.

All analyses were performed stratified by sex and subgroup of supplement use. Unless noted otherwise, *P* < 0.05 was considered statistically significant. Statistical analyses were conducted using spss, version 21 (IBM Corp., Armonk, NY, USA).

## Results

### Proportion of supplement users

In men, 23.1% consumed CLO supplements; however, the prevalences of those taking vitamin A, D and/or E from any supplement sources were 26.5%, 23.2% and 14.8%, respectively (vitamin E was not present, or not quantified by manufacturers in all CLO supplements). Among women, CLO was taken by 26.3% and the prevalence of supplementation with vitamin A, D and/or E was 33.0%, 30.0% and 24.3%, respectively.

### Distribution of food intake and total nutrient intake

**Figure 1:**
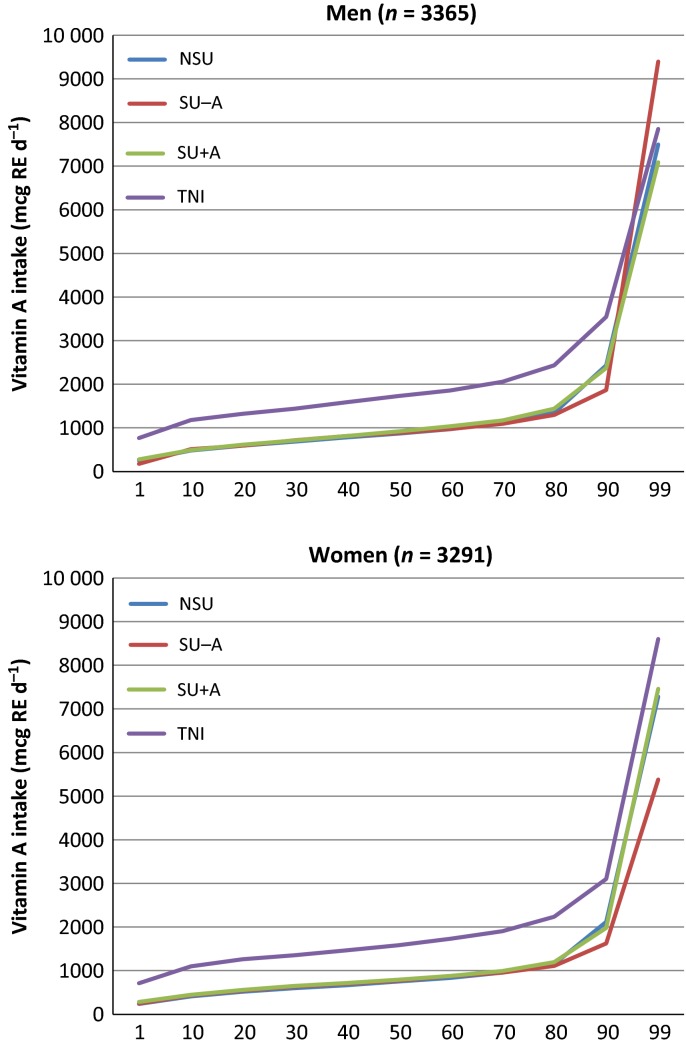
Percentile distribution of vitamin A intake for food sources [nonsupplement user (NSU), supplements without vitamin A (SU-A), supplements with vitamin A (SU+A)] and food and supplement sources (total nutrient intake; TNI). RE, retinol equivalent.

**Figure 2:**
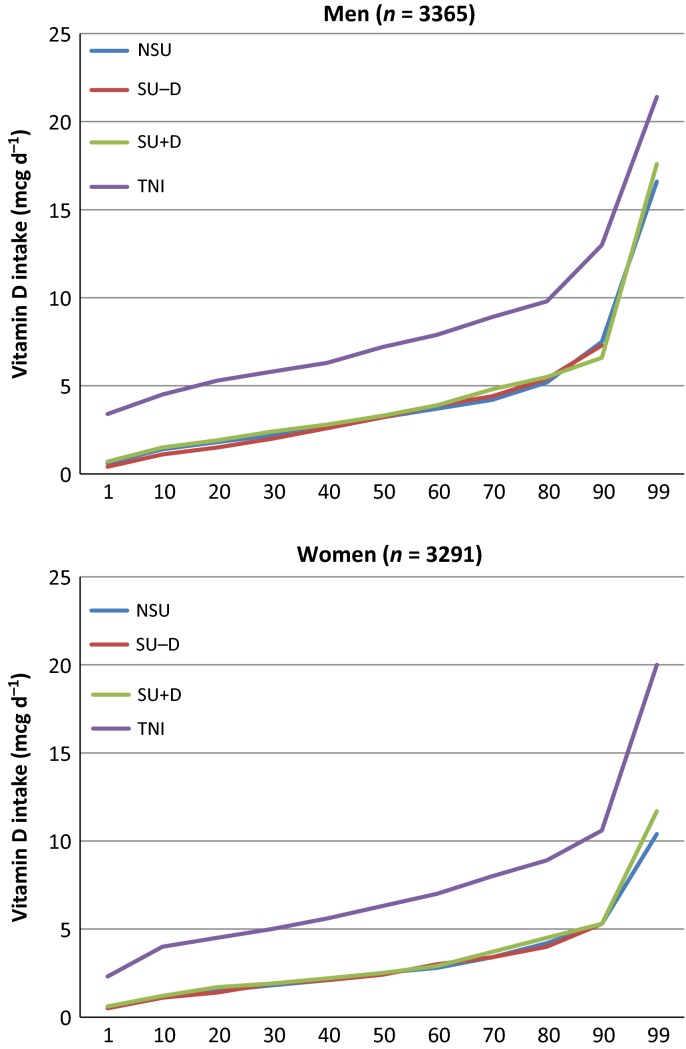
Percentile distribution of vitamin D intake for food sources [nonsupplement user (NSU), supplements without vitamin D (SU-D), supplements with vitamin D (SU+D)] and food and supplement sources (total nutrient intake; TNI).

**Figure 3:**
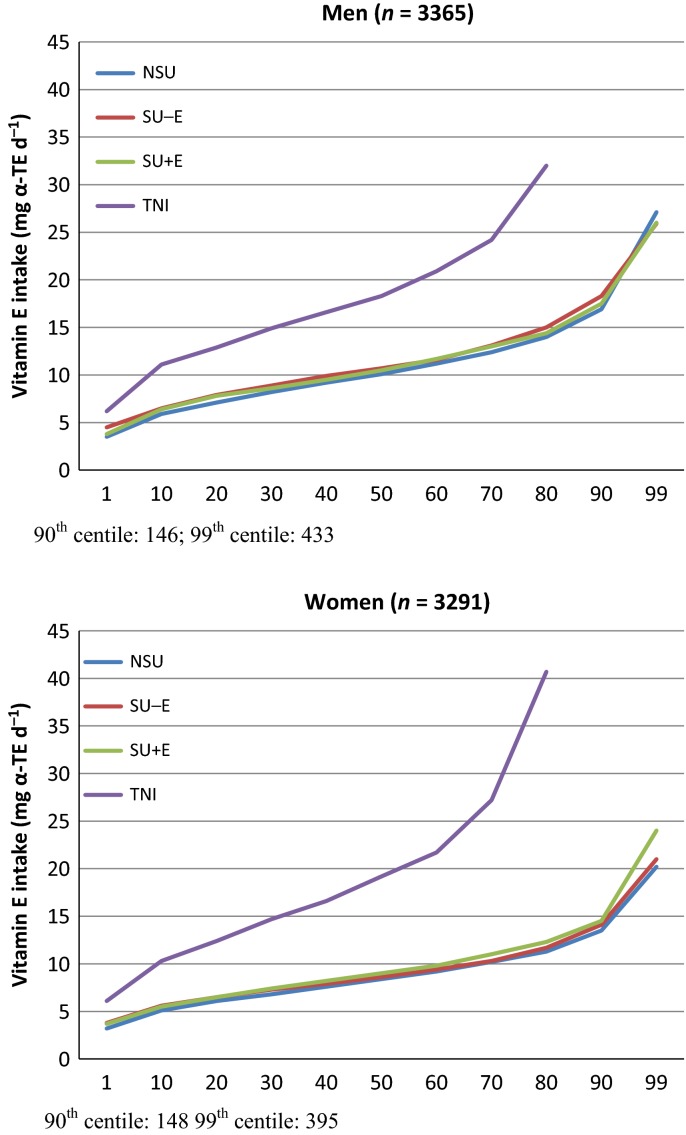
Percentile distribution of vitamin E intake for food sources [nonsupplement user (NSU), supplements without vitamin E (SU-E), supplements with vitamin E (SU+E)] and food and supplement sources (total nutrient intake; TNI). TE, tocopherol equivalent.

**Figure 4:**
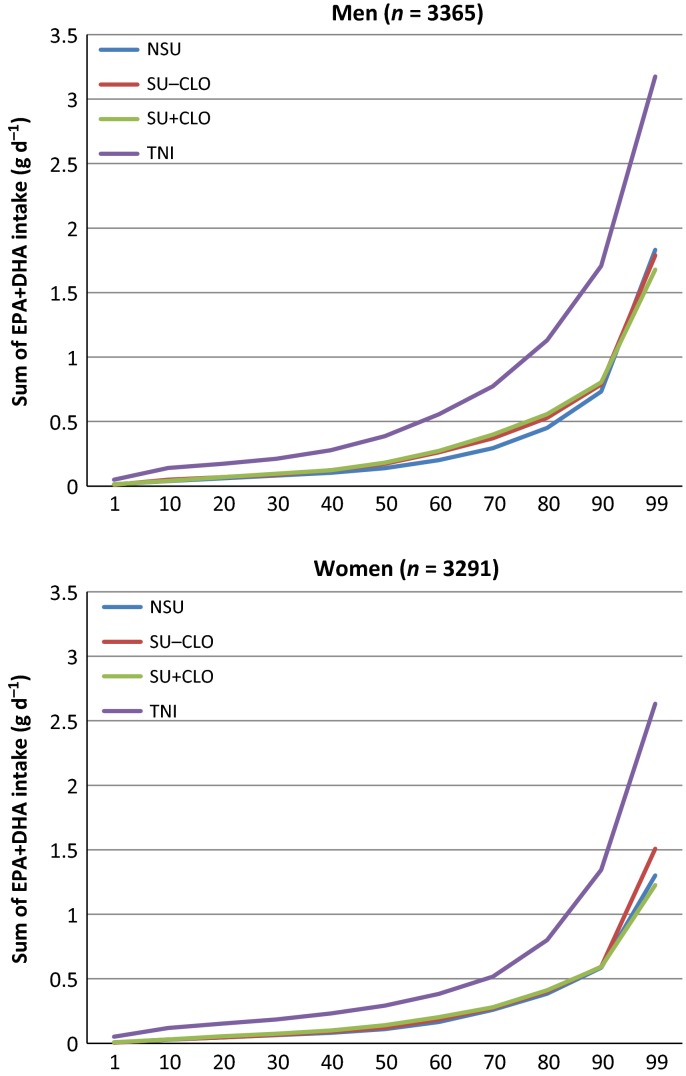
Percentile distribution of eicosapentaenoic acid (EPA) and docosahexaenoic acid (DHA) intake for food sources [nonsupplement user (NSU), supplements without cod liver oil (CLO) (SU-CLO), supplements with CLO (SU+CLO)] and food and supplement sources (total nutrient intake; TNI).

The cumulative percentile distributions of nutrient intake are shown in Figs 1–4. The median supplement dose in the SU+ group for each decile in the food sourced distribution did not show significant differences (data not shown): median supplement intake of vitamin A was 750 μg RE day^−1^; vitamin D was 3.22 μg day^−1^ for men to 2.76 μg day^−1^ for women; vitamin E was 7.1 mg α–TE day^−1^ for men to 9.1 mg α–TE day^−1^ for women; and EPA+DHA was 0.09 g day^−1^. Intake from food sources showed small differences between the three subgroups, most of which were nonsignificant (Table 2). The median TNI in the SU+ subgroup was 1.7- to 2.5-fold higher compared to food sources only. For SU+A, the increase in TNI led to a lower proportion of participants not meeting the EAR and increased the proportion of participants exceeding the GL from approximately 7–12% to 28.1% for men and 23.6% for women. When only considering preformed retinol from supplement sources, these percentages were 5.4% and 5.5% respectively. Only a few participants exceeded the SUL or GL for the other CLO-related vitamins studied.

**Table 2:** Nutrient intake of food and food and supplement (total) sources, proportions not meeting recommended intake or exceeding upper limits, as well as respective plasma concentrations for nonsupplement users (NSU) and subgroups of supplement users (SU)

	NSU				SU−				SU+					
	*N*	%	Median	IQR	*N*	%	Median	IQR	*N*	%	Median	IQR	NSU versus SU−, *P*	SU− versus SU+, *P*
Men														
Vitamin A	2268	67.4			204	6.1			893	26.5				
Food intake, μg RE day^−1^			869	640–1205			885	644–1182			919	667–1262	0.90	0.26
Total intake, μg RE day^−1^			–				–				1730	1382–2194		
<EAR 500 μg RE day^−1^	253	11.2			17	8.3			1	0.1				
>GL 1500 μg RE day^−1^	364	16.0			25	12.3			582	65.2				
>GL 1500 μg retinol day^−1^	268	11.8			18	8.8			251*	28.1				
Plasma retinol, μmol L^−1^			1.75	1.51–2.04			1.73	1.52–1.99			1.85	1.58–2.14	0.83	0.002
<0.70 μmol L^−1^	2	0.1			0				1	0.1				
Vitamin D	463	70.6			41	6.3			152	23.2				
Food intake, μg day^−1^			3.19	2.06–4.70			3.23	1.71–4.55			3.33	2.13–5.23	0.71	0.43
Total intake, μg day^−1^			–				–				7.22	5.51–9.46		
>SUL 25 μg day^−1^	1	0.2			0				0					
Serum 25(OH)D2, nmol L^−1^			1.50	1.00–2.00			1.50	1.06–1.75			1.25	1.00–2.00	0.64	0.91
Serum 25(OH)D3, nmol L^−1^			59.9	44.9–76.1			63.6	49.4–75.4			66.1	53.3–84.2	0.42	0.26
<25 nmol L^−1^	13	2.8			0				1	0.7				
<40 nmol L^−1^	83	17.9			5	12.2			13	8.6				
<75 nmol L^−1^	342	73.9			31	75.6			96	63.2				
Vitamin E	2268	67.4			601	17.9			496	14.8				
Food intake, mg α-TE day^−1^			10.1	7.6–13.1			10.7	8.4–14.1			10.5	8.2–13.7	<0.001	0.028
Total intake, mg α-TE day^−1^			–				–				18.3	13.9–26.8		
>GL 540 mg α- TE day^−1^	0				0				4	0.8				
α-tocopherol, μmol L^−1^			24.7	20.9–29.3			25.3	21.6–29.9			27.5	23.0–34.1	0.049	<0.001
<12 μmol L^−1^	15	0.7			0				1	0.2				
γ-tocopherol, μmol L^−1^			1.69	1.29–2.23			1.62	1.24–2.11			1.41	0.95–1.86	0.032	0.001
EPA+DHA	2268	67.4			318	9.5			779	23.1				
Food intake, g day^−1^			0.14	0.07–0.36			0.17	0.08–0.45			0.18	0.08–0.47	0.014	0.82
Total intake, g day^−1^			–				–				0.39	0.19–0.91		
Plasma EPA+DHA, μmol L^−1^			256	197–338			264	205–354			324	238–429	0.08	<0.001
Women														
Vitamin A	1852	56.3			354	10.8			1085	33.0				
Food intake, μg RE day^−1^			750	558–1024			773	600–1025			794	605–1068	0.25	0.26
Total intake, μg RE day^−1^			–				–				1583	1320–2062		
<EAR 400 μg RE day^−1^	173	9.3			24	6.8			0					
>GL 1500 μg RE day^−1^	235	12.7			40	11.3			615	56.7				
>GL 1500 μg retinol day^−1^	193	10.4			25	7.1			256*	23.6				
Plasma retinol, μmol L^−1^			1.67	1.43–1.96			1.62	1.39–1.93			1.77	1.53–2.04	0.09	<0.001
<0.70 μmol L^−1^	6	0.3			0				2	0.2				
Vitamin D	438	58.9			83	11.2			223	30.0				
Food intake, μg day^−1^			2.51	1.67–3.77			2.36	1.67–3.58			2.53	1.76–4.15	0.97	0.52
Total intake, μg day^−1^			–				–				6.34	4.71–8.38		
>SUL 25 μg day^−1^	0				0				0					
Serum 25(OH)D2, nmol L^−1^			1.50	1.25–2.00			1.50	1.25–2.00			1.50	1.25–2.00	0.19	0.10
Serum 25(OH)D3, nmol L^−1^			55.3	42.2–71.6			53.7	40.7–73.1			67.1	53.2–82.9	0.58	<0.001
<25 nmol L^−1^	19	4.3			5	6.0			4	1.8				
<40 nmol L^−1^	94	21.5			19	22.9			19	8.5				
<75 nmol L^−1^	344	78.5			66	79.5			136	61.0				
Vitamin E	1852	56.3			638	19.4			801	24.3				
Food intake, mg α-TE day^−1^			8.4	6.5–10.6			8.6	6.9–11.0			9.0	7.0–11.4	0.011	0.12
Total intake, mg α- TE day^−1^			–				–				19.2	13.6–33.8		
>GL 540 mg α- TE day^−1^	0				0				3	0.4				
α-tocopherol, μmol L^−1^			26.2	22.2–30.6			27.5	24.0–32.8			29.6	25.0–36.0	<0.001	<0.001
<12 μmol L^−1^	9	0.5			4	0.6			2	0.2				
γ-tocopherol, μmol L^−1^			1.76	1.33–2.36			1.68	1.24–2.28			1.31	0.89–1.85	0.024	<0.001
EPA+DHA	1852	56.3			573	17.4			866	26.3				
Food intake, g day^−1^			0.11	0.06–0.31			0.12	0.06–0.33			0.14	0.06–0.34	0.31	0.16
Total intake, g day^−1^			–				–				0.29	0.17–0.62		
Plasma EPA+DHA, μmol L^−1^			289	223–373			290	227–394			358	272–469	0.22	<0.001

DHA, docosahexaenoic acid; EPA, eicosapentaenoic acid; EAR, estimated average requirement; GL, guideline; IQR, interquartile range; RE, retinol equivalent; SUL, safe upper limit; TE, tocopherol equivalent; n.s., nonsignificant (Mann–Whitney test with significance level of *P* < 0.017).

* Participants above the GL from only preformed retinol supplement sources comprised 48 (5.4%) men and 60 (5.5%) women.

### Associations between nutrient intake and biomarkers

There were no differences in serum/plasma concentrations between the NSU and the SU− groups (Table 2); however, with the exception of 25(OH)D2 and 25(OH)D3 in men, all nutrient concentrations were up to 10% higher in the SU+ group compared to the SU− group for retinol and α-tocopherol; and up to 25% higher for EPA+DHA and 25(OH)D3 in women, although up to 22% lower for γ-tocopherol. Participants in the SU+D group were less likely to have serum 25(OH)D3 concentrations below the various threshold levels commonly applied to identify hypovitaminosis D. Participants with very low retinol and α-tocopherol concentrations were not common in these subcohorts.

The linear association and partial correlations of vitamin intake with their respective (log-transformed) plasma/serum concentration in the three supplement subgroups are presented in Table 3 (men) and Table 4 (women). Vitamin A and vitamin D intakes were weakly correlated with blood retinol and 25(OH)D3, respectively. In the SU+E group, the partial correlation greatly increased for TNI versus food intake only, explaining minimally 13% more of the variance of plasma tocopherols. For α-tocopherol, the linear coefficients in the NSU group were the highest, predicting a 13% and 16% increase in plasma concentration for every 10-mg vitamin E intake increment in men and women, respectively; however, among SU+E, the linear association weakened for both food (7% and 13%, respectively) and even more so for TNI (1%). A similar, but opposite, effect on partial correlation was observed for γ-tocopherol. This analysis was repeated for participants whose TNI did not exceed 17 mg day^−1^, an intake below which 95% of the variance in plasma can be explained by nutrient intake in vitamin E depleted volunteers (Institute of Medicine, 2000). This restriction resulted in stronger linear associations with α-tocopherol in the SU+E group for TNI in women, at the same time as substantially reducing the partial correlations for TNI (compared to the full TNI range of intake among SU+E). Associations between intake and γ-tocopherol became nonsignificant for men but strengthened in women. For EPA+DHA, the linear association remained similar (2–4% increase in plasma per 100 mg increase in intake) across all subcategories of SU, even when supplement sources were included (TNI).

**Table 3:** Association between nutrient intake from food and from food and supplement sources with their respective plasma concentrations. MEN ONLY

	NSU				SU−				SU+				TNI SU+			
Biomarker	*n*	*r*	*e*^β^	95% CI	*n*	*r*	*e*^β^	95% CI	*n*	*r*	*e*^β^	95% CI	*n*	*r*	*e*^β^	95% CI
Retinol (μmol L^−1^) per 800 μg^−1^ RE intake																
1	2248	0.05	0.01	0.00, 0.02	200	−0.02	−0.00	−0.04, 0.03	881	0.04	0.01	−0.01, 0.03	881	0.06	0.02	−0.00, 0.04
2		0.05	0.01	0.00, 0.02		−0.01	−0.00	−0.04, 0.03		0.04	0.01	−0.01, 0.03		0.07	0.02	0.00, 0.04
4		0.05	0.01	0.00, 0.02		0.03	0.01	−0.03, 0.04		0.04	0.01	−0.01, 0.03		0.07	0.02	0.00, 0.04
25(OH)D3 (nmol L^−1^) per 5 μg^−1^ intake																
1	462	0.03	1.29	−2.39, 4.98	41	−0.04	−1.67	−15.20, 11.85	151	0.02	0.77	−6.68, 8.22	151	0.07	2.50	−3.13, 8.13
2		0.03	1.13	−2.69, 4.94		−0.04	−1.68	−15.35, 11.99		0.01	0.40	−7.37, 8.17		0.07	2.37	−3.46, 8.20
3		0.04	1.40	−2.14, 4.93		−0.03	−1.25	−14.42, 11.92		0.01	0.54	−6.78, 7.85		0.01	0.26	−5.41, 5.93
α-tocopherol (μmol L^−1^) per 10 μg^−1^ TE intake																
1	2248	0.14	1.08	1.06, 1.11	593	0.14	1.08	1.03, 1.13	488	0.02	1.02	0.96, 1.08	488	0.39	1.01	1.01, 1.02
2		0.20	1.14	1.11, 1.18		0.17	1.12	1.06, 1.17		0.08	1.06	0.99, 1.13		0.39	1.01	1.01, 1.02
4		0.23	1.13	1.11, 1.16		0.23	1.13	1.08, 1.18		0.10	1.07	1.01, 1.13		0.46	1.01	1.01, 1.02
α-tocopherol (μmol L^−1^)*per 10 μg^−1^ TE intake maximum of 17 mg TE day^−1^																
1	2034	0.08	1.09	1.03, 1.15	517	0.11	1.14	1.02, 1.27	206	0.11	1.14	0.88, 1.48	206	−0.00	1.00	0.74, 1.34
2		0.14	1.20	1.13, 1.28		0.13	1.19	1.05, 1.34		0.12	1.36	0.88, 1.64		−0.01	0.99	0.72, 1.35
4		0.15	1.17	1.12, 1.23		0.19	1.21	1.10, 1.34		0.15	1.21	0.93, 1.58		−0.05	0.95	0.74, 1.21
γ-tocopherol (μmol L^−1^) per 10 μg^−1^ TE intake																
1	2248	−0.07	0.93	0.90, 0.97	593	−0.08	0.93	0.86, 1.00	488	−0.03	0.96	0.86, 1.07	488	−0.38	0.98	0.97, 0.98
2		−0.06	0.93	0.89, 0.98		−0.05	0.95	0.87, 1.03		−0.03	0.94	0.84, 1.06		−0.38	0.98	0.97, 0.98
4		−0.08	0.92	0.88, 0.96		−0.04	0.95	0.88, 1.04		−0.04	0.95	0.84, 1.07		−0.40	0.98	0.97, 0.98
γ-tocopherol (μmol L^−1^)†per 10 μg^−1^ TE intake maximum of 17 mg TE day^−1^																
1	2034	−0.06	0.91	0.86, 0.97	517	−0.01	0.99	0.87, 1.12	206	0.09	1.17	0.91, 1.50	206	0.02	1.03	0.81, 1.32
2		−0.05	0.91	0.85, 0.98		−0.01	1.02	0.88, 1.17		0.08	1.18	0.87, 1.60		0.01	1.01	0.79, 1.31
4		−0.07	0.89	0.83, 0.95		−0.01	0.99	0.87, 1.13		0.13	1.33	0.98, 1.81		0.04	1.07	0.84, 1.36
EPA+DHA (μmol L^−1^) per 100 μg^−1^ intake																
1	2248	0.31	1.03	1.03, 1.04	314	0.29	1.03	1.02, 1.04	768	0.22	1.03	1.02, 1.03	768	0.45	1.03	1.02, 1.03
2		0.31	1.04	1.03, 1.04		0.30	1.03	1.02, 1.04		0.23	1.03	1.02, 1.04		0.45	1.03	1.02, 1.03
4		0.30	1.03	1.03, 1.04		0.30	1.03	1.02, 1.04		0.22	1.02	1.02, 1.03		0.45	1.03	1.02, 1.03

Data presented are from a linear regression, with plasma concentrations of tocopherol and EPA+DHA being log-transformed (values presented are *e*^β^ to be read as the percentage change in plasma for 1 unit increase in intake).

CI, confidence interval; EPA, eicosapentaenoic acid; DHA, docosahexaenoic acid; NSU, nonsupplement users; SU, supplement users; 25(OH)D3, 25-hydroxy cholecalciferol; RE, retinol equivalent; TE, tocopherol equivalent; TNI, total (food + supplement) nutrient intake; *r*, partial correlation.

Model 1: unadjusted;

Model 2: adjusted for energy (per MJ);

Model 3: adjusted for energy (per MJ), season (spring/summer versus autumn/winter), physical activity (active versus inactive), age (per 5 year) and BMI (per kg m^–2^);

Model 4: adjusted for energy (per MJ), total cholesterol (per mmol L^−1^), BMI (per kg m^–2^), smoking (current versus former/never), alcohol (per 8 g of ethanol) and age (per 5 years).

* Median (IQR) of plasma α-tocopherol for SU+E: 25.9 (21.8–30.2) μmol L^−1^.

† Median (IQR) of plasma γ-tocopherol for SU+E: 1.53 (1.23–2.05) μmol L^−1^.

**Table 4:** Association between nutrient intake from food and from food and supplement sources with their respective plasma concentrations WOMEN ONLY

	NSU				SU−				SU+				TNI SU+			
Biomarker	*n*	*r*	*e*^β^	95% CI	*n*	*r*	*e*^β^	95% CI	*n*	*r*	*e*^β^	95% CI	*n*	*r*	e^β^	95% CI
Retinol (μmol L^−1^) per 800 μg^−1^ RE intake																
1	1824	0.01	0.00	−0.01, 0.02	350	−0.02	−0.01	−0.05, 0.04	1068	−0.00	−0.00	−0.02, 0.02	1068	0.01	0.00	−0.01, 0.02
2		0.02	0.01	−0.01, 0.02		−0.02	−0.01	−0.05, 0.05		0.01	0.00	−0.02, 0.02		0.02	0.01	−0.01, 0.03
4		0.01	0.00	−0.01, 0.01		−0.00	−0.00	−0.04, 0.04		0.00	0.00	−0.02, 0.02		0.02	0.01	−0.01, 0.02
25(OH)D3 (nmol L^−1^) per 5 μg^−1^ intake																
1	433	0.14	7.81	2.56, 13.04	83	0.08	5.39	−9.73, 20.50	223	−0.05	−3.18	−10.98, 4.62	223	0.09	3.65	−1.55, 8.84
2		0.13	7.17	1.78, 12.56		0.09	6.35	−11.24, 19.03		−0.02	−1.39	−9.38, 6.61		0.11	4.40	−0.80, 9.59
3		0.03	5.73	0.55, 10.91		0.03	2.05	−13.45, 17.55		−0.01	−0.52	−8.05, 7.02		0.13	4.57	−0.31, 9.45
α-tocopherol (μmol L^−1^) per 10 μg^−1^ TE intake																
1	1824	0.07	1.06	1.02, 1.09	626	0.05	1.03	0.98, 1.10	792	.04	1.03	0.98, 1.09	792	0.32	1.01	1.01, 1.01
2		0.14	1.14	1.09, 1.19		0.07	1.06	0.99, 1.13		.01	1.09	1.02, 1.16		0.32	1.01	1.01, 1.01
4		0.20	1.16	1.12, 1.19		0.10	1.07	1.01, 1.13		.16	1.13	1.07, 1.19		0.41	1.01	1.01, 1.01
α-tocopherol (μmol L^−1^)* per 10 μg^−1^ TE intake maximum 17 mg TE day^−1^																
1	1766	−0.05	1.04	1.00, 1.09	604	0.06	1.06	0.98, 1.14	329	−0.03	0.96	0.86, 1.08	329	0.06	1.06	0.96, 1.17
2		0.12	1.15	1.09, 1.21		0.08	1.09	1.00, 1.19		0.05	1.06	0.92, 1.23		0.10	1.10	0.89, 1.21
4		0.14	1.17	1.12, 1.22		0.11	1.09	1.02, 1.17		0.13	1.14	1.02, 1.27		0.17	1.13	1.04, 1.22
γ-tocopherol (μmol L^−1^) per 10 μg^−1^ TE intake																
1	1824	−0.08	0.91	0.85, 0.96	626	−0.09	0.89	0.81, 0.99	792	−0.06	0.92	0.83, 1.01	792	−0.40	0.97	0.97, 0.98
2		−0.05	0.93	0.87, 1.00		−0.05	0.92	0.82, 1.04		−0.06	0.90	0.80, 1.02		−0.40	0.97	0.97, 0.98
4		−0.04	0.95	0.89, 1.01		−0.05	0.93	0.83, 1.03		−0.04	0.94	0.83, 1.05		−0.40	0.97	0.97, 0.98
γ-tocopherol (μmol L^−1^)† per 10 μg^−1^ TE intake maximum 17 mg TE day^−1^																
1	1766	−0.11	0.84	0.79, 0.91	604	−0.09	0.87	0.77, 0.99	329	0.02	1.04	0.85, 1.28	329	−0.12	0.83	0.70, 0.99
2		−0.08	0.86	0.79, 0.94		−0.05	0.91	0.78, 1.05		−0.00	0.99	0.77, 1.27		−0.13	0.81	0.68, 0.96
4		−0.07	0.89	0.82, 0.96		−0.06	0.91	0.80, 1.04		0.04	1.08	0.86, 1.37		−0.13	0.83	0.70, 0.97
EPA+DHA (μmol L^−1^) per 100 μg^−1^ intake																
1	1824	0.27	1.04	1.03, 1.04	566	0.29	1.04	1.03, 1.05	852	0.16	1.02	1.01, 1.04	852	0.37	1.03	1.02, 1.03
2		0.28	1.04	1.03, 1.05		0.30	1.04	1.03, 1.05		0.16	1.02	1.01, 1.04		0.37	1.03	1.02, 1.03
4		0.26	1.04	1.03, 1.04		0.30	1.04	1.03, 1.05		0.15	1.02	1.02, 1.03		0.38	1.03	1.02, 1.03

Data presented are from a linear regression, with plasma concentrations of tocopherol and EPA+DHA being log-transformed (values presented are *e*^β^ to be read as the percentage change in plasma for 1 unit increase in intake). CI, confidence interval; EPA, eicosapentaenoic acid; DHA, docosahexaenoic acid; NSU, nonsupplement users; SU, supplement users; 25(OH)D3, 25-hydroxy cholecalciferol; RE, retinol equivalent; TE, tocopherol equivalent; *r*, partial correlation. Model 1: unadjusted.

Model 2: adjusted for energy (per MJ).

Model 3: adjusted for energy (per MJ), season (spring/summer versus autumn/winter), physical activity (active versus inactive), age (per 5 years) and BMI (per kg m^–2^);

Model 4: adjusted for energy (per MJ), total cholesterol (per mmol L^−1^), BMI (per kg m^–2^), smoking (current versus former/never), alcohol (per 8 g of ethanol) and age (per 5 years).

* Median (IQR) of plasma α-tocopherol: 27.7 (23.9–33.4) μmol L^−1^.

† Median (IQR) of plasma γ-tocopherol: 1.53 (1.08–2.13) μmol L^−1^.

**Figure 5:**
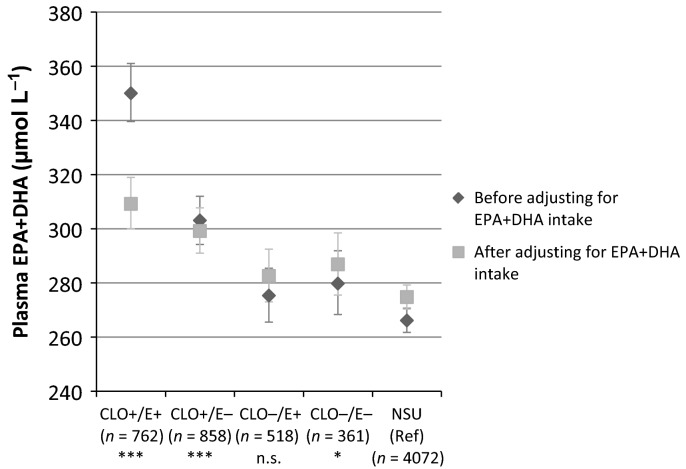
Estimated marginal means and 95% confidence interval for plasma eicosapentaenoic acid (EPA) + docosahexaenoic acid (DHA) by use of cod liver oil (CLO) and/or vitamin E supplements. The F-test for interaction between CLO and vitamin E became nonsignificant after inclusion of total nutrient intake (TNI) EPA+DHA intake as a continuous variable (per 100 mg) (P = 0.129). ^a^Means were adjusted for cholesterol, age, body mass index, alcohol consumption, smoking and sex. *P < 0.05; *** P < 0.001; n.s., nonsignificant (Wald test, after adjusting for TNI). NSU, nonsupplement users; E, vitamin E.

Finally, we tested for the interaction between vitamin E and CLO supplement use and found that mean EPA+DHA concentrations were significantly higher if vitamin E was supplemented together with EPA+DHA compared to EPA+DHA only (Fig.5); however, its significance disappeared (*P* = 0.13) after adjusting for TNI, indicating that the higher dose of EPA and DHA in vitamin E containing CLO was mainly driving the higher plasma EPA+DHA concentration.

## Discussion

Nutrients related to CLO consumption were supplemented by 15% to 33% of the participants. Inclusion of supplements resulted in approximately a doubled nutrient intake. TNI of vitamin A reduced the proportion below the EAR but also increased the proportion above the GL. Partial correlations between intake and biomarkers improved after the inclusion of supplement sources but vitamin E intake exceeding food intake resulted in attenuated coefficients, indicating minimal to no further increase in the concentrations of the biomarkers studied. This association was not observed for plasma EPA+DHA concentrations.

We observed an upward shift of 0.1 μmol L^−1^ in the distribution of plasma retinol for the SU+A group versus NSU and SU-A. The partial correlations between intake and plasma were small, even among SU+A users. This is in line with a review indicating that plasma retinol concentrations are not responsive to hypervitaminosis of vitamin A (Penniston & Tanumihardjo, 2006). The differential response of α- and γ-tocopherol on vitamin E supplementation has been found in clinical trials (Yoshikawa *et al*., 2005; Clarke *et al*., 2006). Supplements can contain a mixture of tocopherols, although α-tocopherol tends to dominate. α-Tocopherol transfer protein has preferential incorporation of α-tocopherol into lipoproteins, which leads to increased metabolites of γ-tocopherol and hence a reduction of plasma γ-tocopherol. A meta-analysis of dose–response studies with either EPA or DHA suggests that intakes of up to 4 g and 2 g, respectively, show a clear linear association with biomarker data; however, when supplemented together, this association becomes less strong and only linear below 1.2 g day^−1^ (Arterburn *et al*., 2006). In this observational study, we found a weak but consistent association across ranges of intakes from food and TNI that only in a small proportion exceeded 1.2 g day^−1^.

For the selection of nutrients studied, we found small and mainly nonsignificant differences between the different subgroups in intake distributions from food sources only. Results from the UK Women's Cohort Study have shown that food nutrient intake among SU was higher than among NSU (Kirk *et al*., 1999); however, the results were obtained with a food frequency questionnaire and are not suitable for comparison against diet reference intakes. The British Birth Cohort used a 5-day diet diary and observed that, even after adjustment for social class, area of residence, smoking, alcohol and physical activity, SU were proportionately more likely to be in the upper tertile of food nutrient intake compared to NSU (McNaughton *et al*., 2005). A comparison of the EPIC-Norfolk nutrient intake with the 50–64-year-old category of the 2000/2001 NDNS report (Henderson *et al*., 2003) and 65–74-year-old category of the 1998 report (Finch *et al*., 1998) show that the median food intakes of the fat-soluble vitamins in EPIC-Norfolk were lower, with the exception of vitamin A. The proportion estimated to have *food* nutrient intake above 1400 μg RE day^−1^ is 18% among men and 8% among women; intakes above 1600 μg RE day^−1^ are found for 13% and 7%, respectively (Henderson *et al*., 2003). This range approximates the 16.0% found in men in EPIC-Norfolk exceeding 1500 μg RE day^−1^, although we found a higher prevalence (12.7%) among women. Both food and supplement sources are able to exceed the GL intake and therefore we have presented excess intakes for the subgroups separately. We observed that 24–28% of the SU+A users had a preformed retinol intake that exceeded the GL level and could be at increased risk of bone fractures (Expert Group on Vitamins and Minerals, 2003). In the 2003–06 data from the American National Health And Nutrition Examination Survey (NHANES), where only preformed retinol from supplement sources is considered a possible risk, this percentage is 4–5% (Bailey *et al*., 2012). The proportion of participants found at risk according to this definition was similar for EPIC-Norfolk.

We quantified the association between intake and biomarker using both partial correlation and coefficients from linear regression. Correlations between TNI and plasma tocopherol found in this study were lower compared to those found in other cohorts (White *et al*., 2001; Satia-Abouta *et al*., 2003). This might be explained by the higher proportion of high-dose (>400 mg day^−1^) vitamin E in these American cohorts, increasing α-tocopherol by 84% and decreasing γ-tocopherol by 72% (White *et al*., 2001). Considering the median supplement dose in EPIC-Norfolk is below 10 mg day^−1^ and because the correlation coefficient is dependent on the range of the input variables, we restricted the intake to a range known to be linearly associated with plasma concentrations (Institute of Medicine, 2000) and found that, among men, the intake of food sources (NSU, SU-E), rather than TNI, was more strongly associated with plasma tocopherols. Among women, linear associations strengthened after the inclusion of supplement sources. In line with White *et al*. (2001), we found lower γ-tocopherol concentrations at relatively low vitamin E intakes (<17 mg day^−1^); this lower concentration might reduce the anti-inflammatory and anti-oxidant function of γ-tocopherol (Jiang *et al*., 2001). Although we did not find strong linear associations between vitamin D intake and serum 25(OH)D, among SU+D we observed higher median 25(OH)D3 concentrations and lower proportions of participants below the tested hypovitaminosis thresholds. Also in the British Birth Cohort, it was found that vitamin D supplements reduced the risk of not attaining concentrations of 40 nmol L^−1^ (Hyppönen & Power, 2007).

In this analysis, statistical adjustment of the nutrient distributions obtained was not attempted (National Cancer Institute, 2011). We acknowledge that more than 7 days of diet collection are necessary to obtain a precise estimate of nutrient intake, particularly for vitamin A (Nelson & Bingham, 1997). However, the main aim of EPIC-Norfolk is to establish associations between diet and chronic disease. The statistical methods applied for this purpose often rely on ranking and less on absolute intakes and so the inclusion of supplement sources, which will move SU to a higher rank, will have a stronger influence on the results than narrowing the distribution. Nevertheless, measurement error could have overestimated the proportions at risk of excessive intake. Although diary data in our cohort can estimate α- or γ-tocopherol separately, many labels on manufacturers’ supplements were not sufficiently detailed to establish the source; hence, we were unable to ascertain the effects of the individual vitamin E forms. Similar issues arose for vitamin D_2_ and vitamin D_3_. For 75% of the supplements in the ViMiS database, the form of vitamin D was not given by the manufacturers; however, vitamin D found in CLO can be assumed to be D_3_. Moreover, we did not observe a decrease in serum 25(OH)D3, which has been shown to occur when supplemented with D_2_ (Logan *et al*., 2013). Manufacturer's use of overages (Expert Group on Vitamins and Minerals, 2003), where a higher dose than stated on the packaging can be found in the supplement consumed, could also have obscured our findings. Lastly, our results were limited to subsets of the EPIC-Norfolk population and, particularly for the vitamin D subset, the number of participants in each supplement subgroup was limited, ensuing wide confidence intervals for estimates.

Although intake in the SU+A group reduced the proportion of participants not meeting the EAR to almost 0%, it increased daily intake above GL levels, which has been associated with adverse health outcomes such as lower bone density (Expert Group on Vitamins and Minerals, 2003). ‘More is better’ does not apply to all nutrient intakes when related to their blood biomarker. Higher intakes of the fat-soluble vitamins A and D were unrelated to biomarker status and vitamin E at intakes above 17 mg day^−1^ became weakly associated with α-tocopherol concentrations. From this perspective, there does not appear to be any benefit of high-dose supplements, particularly for vitamin E, because ingestion of large quantities of α-tocopherol (the type mostly found in supplements) can decrease γ-tocopherol concentrations, which might reduce anti-inflammatory capacity (Jiang *et al*., 2001). Moreover, a meta-analysis has shown an increased risk of mortality when supplementing vitamins A and E (Bjelakovic *et al*., 2008).

## Conclusions

From these results, we conclude that nutrient intake of vitamins A, D and E, as well as EPA and DHA, is approximately doubled when subdividing SU into SU+ and SU− subgroups. However, although plasma concentrations tend to be higher among SU+, the linear associations (with the exception of EPA+DHA) are inconsistent across the supplement subgroups, indicating that the supplement itself had a minimal contribution to blood concentrations.

**Conflict of interests, sources of funding and authorship**

The authors declare that they have no conflict of interests.

All authors report receiving grants from Cancer Research UK and grants from the Medical Research Council (MRC) during the study. The MRC and Cancer Research UK had no role in the design, analysis and writing of the article.

MAHL prepared the manuscript; analysed the data; cleaned and calculated the 7dDD data; assisted in the programming of the 7dDD processing software ‘dinermo’, and developed the concepts for the ViMiS calculation software and extended database. AAM cleaned and calculated the 7dDD data and contributed to the early work on the dietary supplement data, as well as the development of the original database. AB wrote the ViMiS software. AAW developed the data-entry software for the 7dDD (diner); instigated the original database of supplements; and contributed to the early work on the dietary supplement data and oversaw its later developments. RNL is responsible for overall data management and contributed to the development of the original database. KTK is the principal investigator of the EPIC-Norfolk study. All authors critically read and contributed to the manuscript that was submitted for publication.

## Supporting information

**Figure S1.** The association between nutrient intake and plasma concentrations stratified by sex and grouped by supplement subgroup. Note that the *x*-axis is portrayed on a log_10_ scale, whereas numbers refer to the absolute, or ‘natural’, intake.
