# Supplementary material for: Contribution of cod liver oil-related nutrients (vitamins A, D, E and eicosapentaenoic acid and docosahexaenoic acid) to daily nutrient intake and their associations with plasma concentrations in the EPIC-Norfolk cohort
Source: J Hum Nutr Diet. 2014 Sep 16;28(6):568–82. doi: 10.1111/jhn.12271 (PMC4657496; doi:10.1111/jhn.12271)

Supplement 1: The association between nutrient intake and plasma concentrations stratified by sex and grouped by supplement subgroup. Please note that the x-axis is portrayed on a log10-scale, but the numbers refer to the absolute, or ‘natural’, intake.


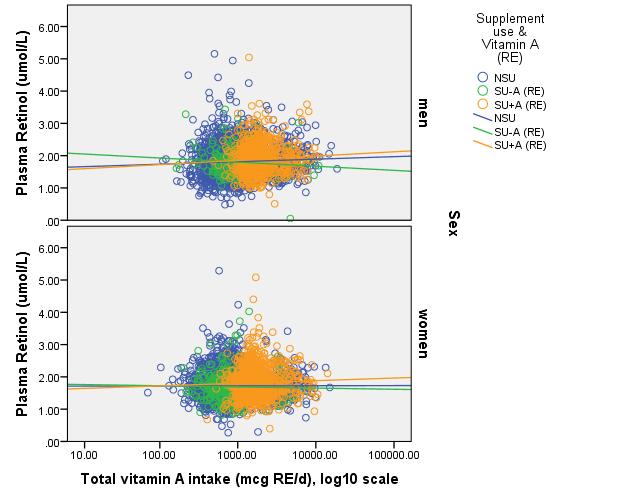


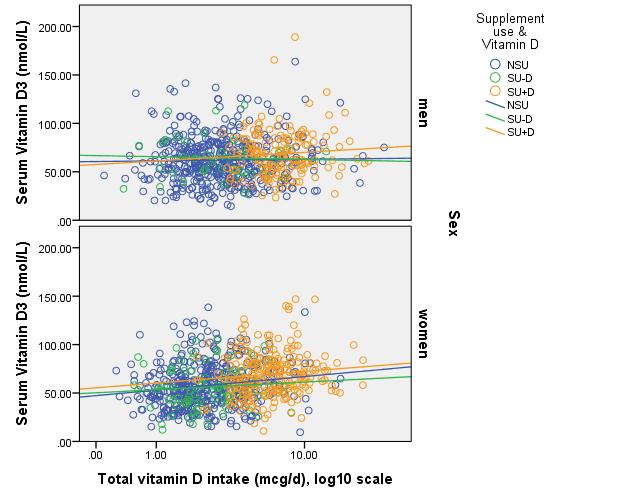

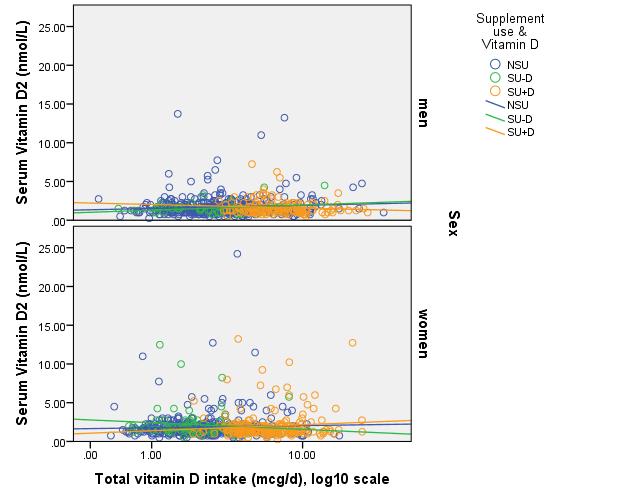


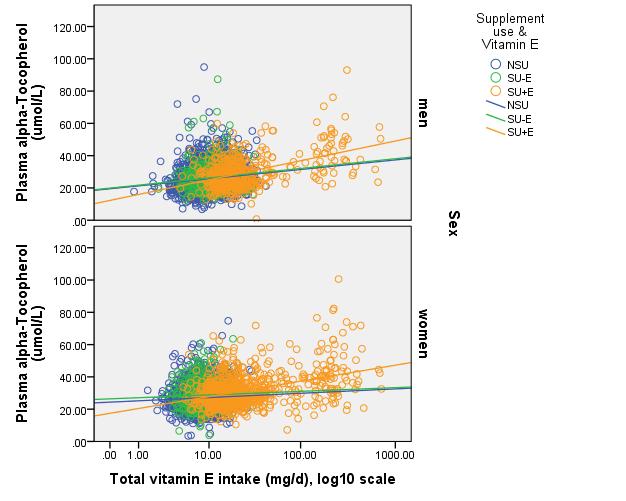

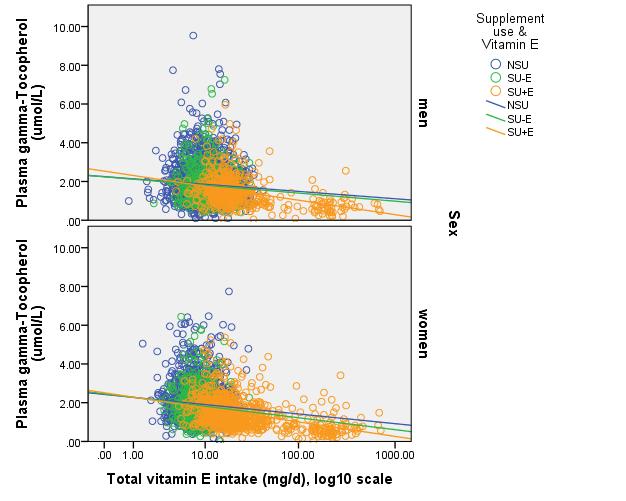


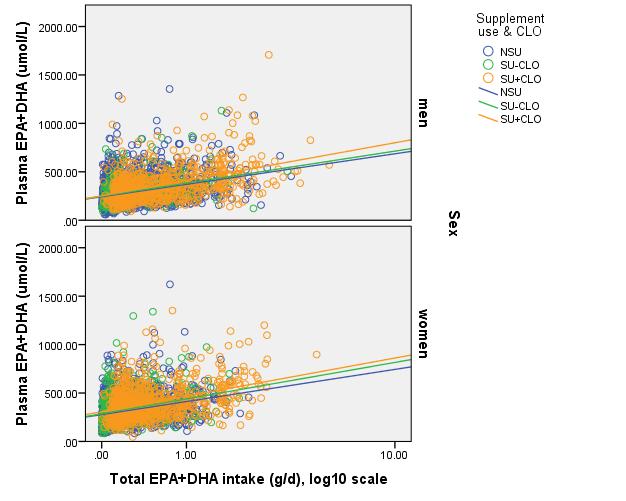

Supplement: Supplementary file 1 — Figure S1. The association between nutrient intake and plasma concentrations stratified by sex and grouped by supplement subgroup. Note that the x-axis is portrayed on a log10 scale, whereas numbers refer to the absolute, or ‘natural’, intake. [file jhn0028-0568-sd1.docx]
